# Splitting of standing spin-wave modes in circular submicron ferromagnetic dot under axial symmetry violation

**DOI:** 10.1038/srep18480

**Published:** 2015-12-22

**Authors:** S. A. Bunyaev, V. O. Golub, O. Yu. Salyuk, E. V. Tartakovskaya, N. M. Santos, A. A. Timopheev, N. A. Sobolev, A. A. Serga, A. V. Chumak, B. Hillebrands, G. N. Kakazei

**Affiliations:** 1 IFIMUP and IN-Institute of Nanoscience and Nanotechnology, Departamento de Física, Universidade do Porto, 4169-007 Porto, Portugal; 2 Institute of Magnetism, National Academy of Sciences of Ukraine, 36b Vernadskogo Blvd, 03142 Kiev, Ukraine; 3 Departamento de Física and I3N, Universidade de Aveiro, 3810-193 Aveiro, Portugal; 4 National University of Science and Technology “MISiS”, 119049 Moscow, Russia; 5 Fachbereich Physik and Forschungszentrum OPTIMAS, Technische Universität Kaiserslautern, 67663 Kaiserslautern, Germany

## Abstract

The spin wave dynamics in patterned magnetic nanostructures is under intensive study during the last two decades. On the one hand, this interest is generated by new physics that can be explored in such structures. On the other hand, with the development of nanolithography, patterned nanoelements and their arrays can be used in many practical applications (magnetic recording systems both as media and read-write heads, magnetic random access memory, and spin-torque oscillators just to name a few). In the present work the evolution of spin wave spectra of an array of non-interacting Permalloy submicron circular dots for the case of magnetic field deviation from the normal to the array plane have been studied by ferromagnetic resonance technique. It is shown that such symmetry violation leads to a splitting of spin-wave modes, and that the number of the split peaks depends on the mode number. A quantitative description of the observed spectra is given using a perturbation theory for small angles of field inclination from the symmetry direction. The obtained results give possibility to predict transformation of spin wave spectra depending on direction of the external magnetic field that can be important for spintronic and nanomagnetic applications.

An analytical description of spin waves in patterned magnetic structures is a complicated problem. Due to the inhomogeneity of the internal demagnetizing field, it is impossible to find exact eigenfunctions for most of the geometries. The good agreement between analytical calculations and experimental data was found for only few cases of simplest symmetry, for example, infinite stripes magnetized along two principal directions^1^,^2^ and axially magnetized circular dots^3^. In the former case, it was natural to assume that the eigenfunctions of the stripes would have an almost sinusoidal form, analogous to the form of spin wave resonance modes in a perpendicularly magnetized continuous homogeneous film. In the latter case, the spin wave profiles were satisfactorily described by zero-order Bessel functions. In both cases the demagnetizing fields for different modes were calculated with relative easiness. However, for practical applications of patterned elements, i.e. in read-write magnetic heads, the direction of the external magnetic field may deviate from the symmetry axis. In such devices additional spin-wave modes can be considered as magnetic noise^4^. Therefore, it is important to investigate the evolution of the spin wave spectra in the case of a symmetry violation.

In this paper we study, both experimentally and theoretically, the modification of such spectra for circular Permalloy (Ni_81_Fe_19_) dots in external magnetic fields slightly inclined from the perpendicular direction. For this orientation the most efficient experimental techniques are either cavity-based^3^,^5^,^6^,^7^,^8^ or broadband^9^ ferromagnetic resonance (FMR) spectroscopy as well as FMR force microscopy^10^,^11^. Here the well established cavity-based X-band FMR was applied, that allows to probe the millimeter-size samples and to use sample holder with both in-plane and out-of-plane angular rotations.

## Results

### Sample design

**Figure 1:**
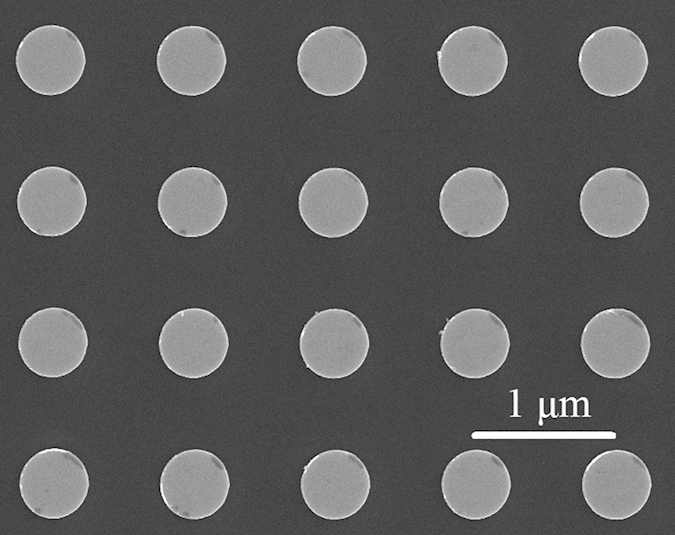
Scanning electron microscopy image of Permalloy dot array under study. The dot radius is 250 nm, center-to-center interdot distance is 1000 nm and dot thickness is 40 nm.

A square array of circular Py dots was prepared on a Si wafer with an oxidized surface Since the distance between the neighbour resonance peaks of standing spin waves in a perpendicularly magnetized circular dot is inversely proportional to the dot radius *R*^3^, we selected *R* to be 250 nm. In this case the shape of the dot is very close to the circular (as confirmed by scanning electron microscopy after removing the resist, see Fig. 1) and the distance between the resonance peaks is large enough to study the splitting in a broader angle range. The interdot center-to-center distance was equal to 4*R* = 1000 nm, which is sufficient to exclude the influence of interdot magnetostatic interactions on the resonance peak positions. The total size of the array was 2000 × 2000 elements, sufficient to obtain spin-wave spectra with a signal-to-noise ratio >100 even for the 5^th^ peak. After resist development Permalloy (Py, Ni_80_Fe_20_) film with a thickness *L* = 40 nm was deposited. Finally after lift-off process the array of circular Py dots with well-pronounced edges was formed on Si surface.

### Spin wave spectroscopy

**Figure 2:**
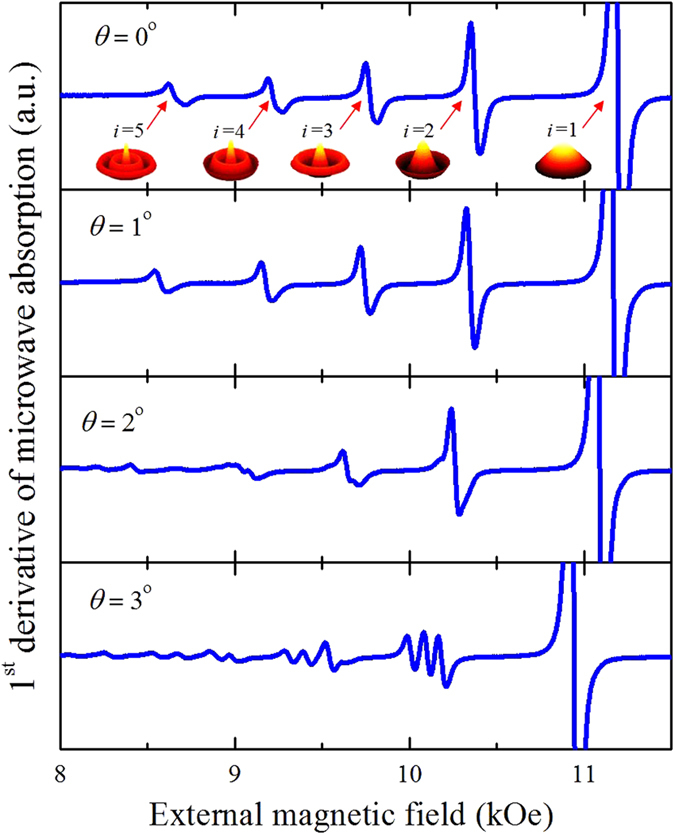
Evolution of the 1^st^ derivative of the spin-wave spectra of an array of circular dots with increase of angle *θ*. The main mode with index *i* = 1 was partially cut to make all other modes clearly visible. Inserts show zero-order Bessel-function profiles for corresponding circular “drumhead” modes.

We started our measurements with a precise orientation of the array under study with respect to the external magnetic field. To do so, the angular dependence of the resonance field for the most intense peak was measured in the vicinity of the normal as a function of *θ*. The maximum value of *H*_res_ corresponded to the perfect alignment of *n* along *H*. For this geometry, five sharp resonance peaks with their intensities growing with increasing resonance field were clearly observed (see Fig. 2, *θ* = 0°). This spectrum is similar to the one observed in ref. [3] where profiles of these standing spin waves were satisfactorily described by zero-order Bessel functions. Resonance fields and interpeak distances are a bit different from those observed in^3^ since they depend on the dot radius and thickness, in full agreement with analytical theory.

**Figure 3:**
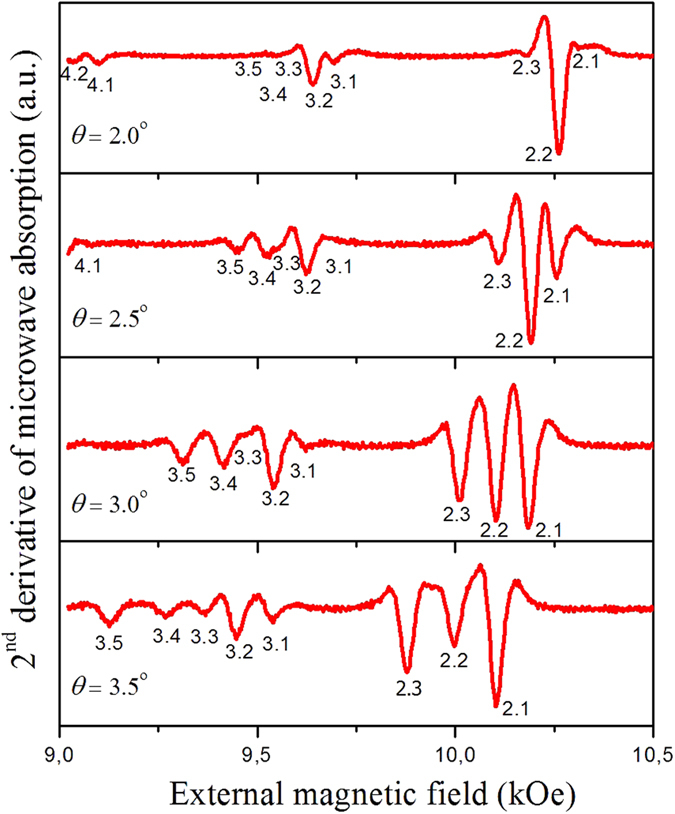
Evolution of the 2^nd^ derivative of the spin-wave spectra of an array of circular dots with increasing angle between the normal to the sample surface and direction of the external magnetic field. The peaks of the spin wave modes are numbered as *i.s*, where *i* is the mode number and *s* is level number. The first mode *i* = 1 is not shown on the plot.

**Figure 4:**
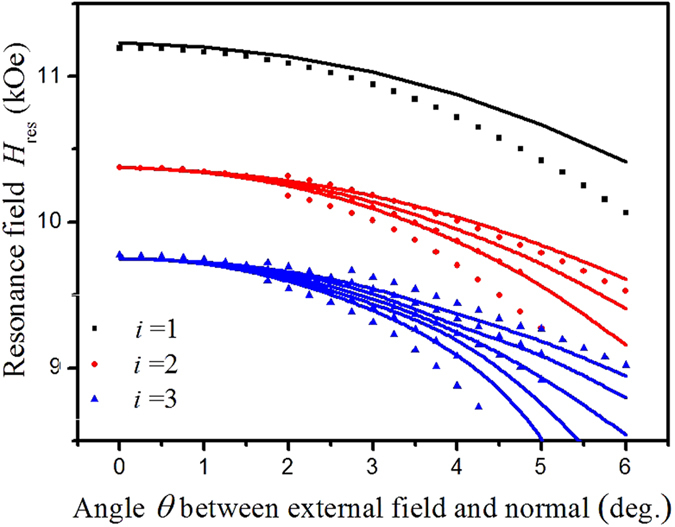
Dependence of the resonance fields of the spin wave modes (numbered by *i*) on the angle between the normal to the sample surface and direction of the external magnetic field. Points represent the experimental data, lines show theoretical fits.

Then a series of spin wave spectra were measured from *θ* = 0^o^ to 5^o^ with an increment of 0.25^o^. It can be assumed from Fig. 2 that the modes are starting to split with angle *θ* inclination from the normal, however, the features of the splitting process are not pronounced enough. Therefore to clarify the number of split peaks for each particular mode and to track their resonance fields, the following procedure was introduced. First, the measured FMR spectrum (it is the 1^st^ derivative of the microwave absorption) was integrated, then the 1^st^ order low-pass Butterworth filter^12^ was applied to correct the signal baseline, and finally the 2^nd^ derivative was calculated to increase the accuracy of the resonant field determination of the split modes. As a result, we found the following numbers of split peaks: three for the second mode, five for the third one (see Fig. 3). Starting from the 4^th^ mode the separation process is affected by the overlap between peaks of the several modes. The obtained dependencies of the resonance fields of the spin wave modes (numbered as ***i.s***, where ***i*** is the mode number (*i* = 1, 2, …) and ***s*** is the level number) on the angle between the normal to the sample surface and direction of the external magnetic field are presented in Fig. 4.

The results shown in Figs 3 and 4 can be summarized in the following way: a) If *θ* ≠ 0, all resonance peaks except for the main one are split, i.e. all modes are split even for the very small angles *θ* but it becomes evident only with increase of *θ* when the splitting distance is large enough. For small angles the splitting looks just like broadening of the resonance line; b) the splitting value is proportional to the mode number and angle *θ*; c) the number of the split peaks depends on the mode number as 2*i* – 1.

### Analytical theory

To explain abovementioned systematic behavior, the extension of the analytical theory presented in ref. [3] is proposed here. We start from the linearized Landau–Lifshitz equation for the components of the variable magnetization ***m*** perpendicular to the static magnetization ***M***^13^,^14^,


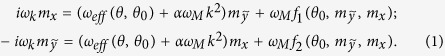


**Figure 5:**
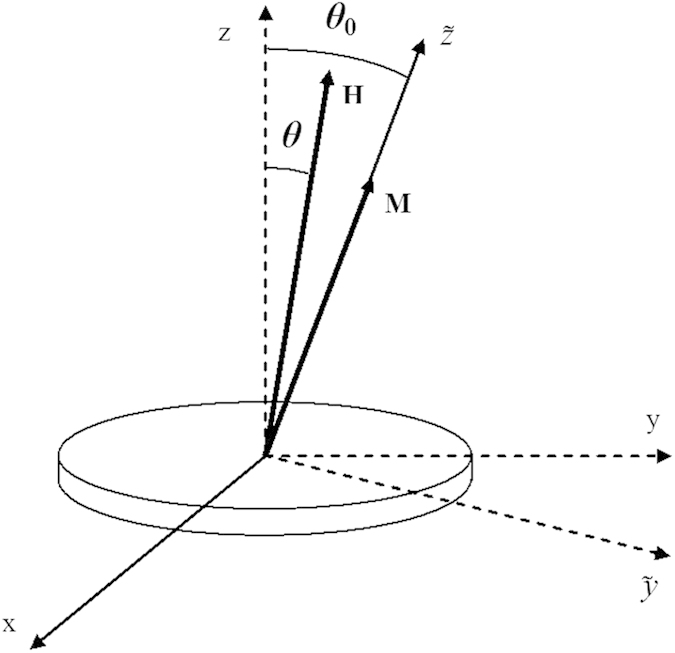
Coordinate system used in the theoretical calculations.

Here *ω*_*k*_ is the spin wave (SW) frequency, ***k*** is the radial wave vector of the SW, *ω*_*M*_ = γ4π*M*. We assume the external field ***H*** and the magnetization ***M*** to be confined in the *zy* plane. 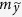 is a component of the variable magnetization along the 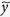 axis (Fig. 5) of the canted coordinate system (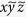) with the new 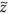 axis directed along the ***M*** vector (see refs [13, 14, 15]). The angle between ***H*** and the normal to the dot plane (*0z* axis) is *θ*, the angle between ***M*** and the normal to the dot plane, *θ*_0_, can be easily calculated from the balance between the Zeeman and magnetostatic energies. The matrix elements of the dipole-dipole interaction have the form:


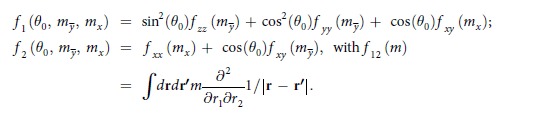


The exchange constant 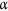 and the effective matrix elements *N*_*m*_ of the inhomogeneous demagnetizing fields for different standing spin wave modes are defined in the same way as in ref. [3]: 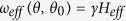, where 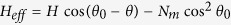 is an analogue of the effective bias magnetic field in the case of a deviation of the direction of the external magnetic field from the symmetry axis.

The expression for *H*_*eff*_ is a sum of two terms - the components of the external field and of the static demagnetizing field along the M direction, like in ref. 14. However, the calculation of the components of the dipole-dipole tensor that we describe below is quite different from the presented in^14^. As it is known, both full sets - Bessel functions and plain waves - are not revealed as the sets of exact eigenfinctions of the dipole-dipole tensor. It was shown in^3^ that for the case of thin perpendicularly magnetized circular disks the expression derived previously for the dipolar-dipolar tensor in thin films^13^ is the most reliable approximation. Since we consider here the splitting of modes due to the small deviation from the perpendicular geometry, it is convenient and physically reliable to use the same approach as a starting point of the perturbation theory, presented below. We underline that this approach is valid only for the case of small inclination angles, while for larger angles the theory presented in^14^ is more consistent and reliable. So, the physical objects described here and in^14^ are similar, while the physical effects considered in our work and in^14^ are different. We are inclined to determine this as two mutually complementary investigations of the magnetic properties of thin circular nanodisks. It is worth to note that both descriptions are not suitable for the case when the external field angle *θ* is approaching π/2. In the latter case the excitation spectra includes the modes confined near the dot ages^16^.

In the framework of the perturbation theory (i.e. when the *θ*_0_ value is small), we substitute dipolar operators in the system (1) with c-numbers:


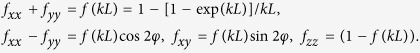


A similar substitution was performed in ref. 3 for the case of a perpendicular field. In such a case the system of two integral equations (1) can be reduced to the expression:


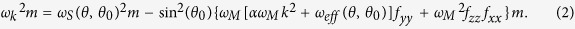


For the case *θ* = *θ*_0_ = 0, the term


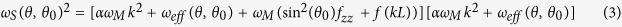


coincides with the formula for spin-wave spectra (modified Kittel formula) for perpendicularly magnetized circular dots^3^. The eigenfunctions satisfying the dipolar infinite “pinning” boundary conditions can be written as 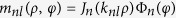, where 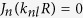, *φ* is an axial angle of the SW excitations vector. In the following we assume that the quantized values of the spin-wave vector satisfy the abovementioned conditions, i.e. *k* = *k*_*nl*_, where *n* corresponds to the index of the Bessel functions while *l* is a number of its root. To find the experimentally observed SW frequencies we substitute the expansion of the variable magnetization ***m*** by eigenfunctions with even *n* and average the expression over the 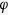 angles.

**Figure 6:**
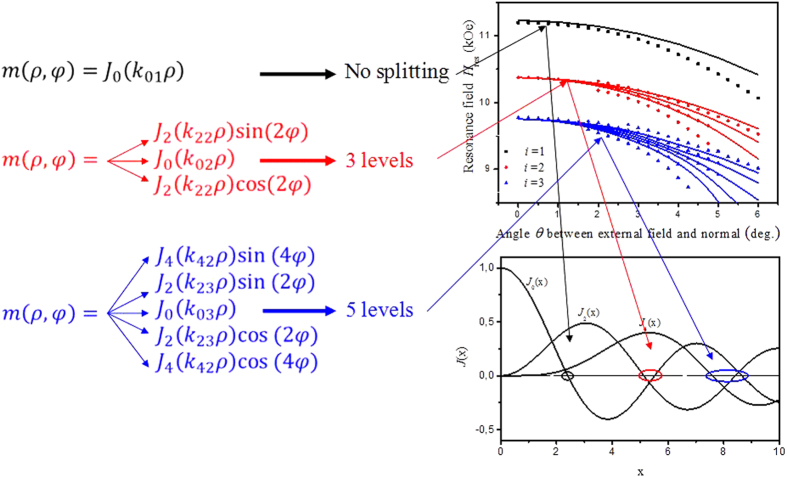
Explanation of the dependence of the number of split peaks on the mode number. The left panel: analytical expressions of the profiles for three first modes. The lowest mode (black letters) is not degenerated in the case of perpendicular field. The second mode (red letters) contains the radially symmetrical Bessel eigenfunction (n′ = 0) and two Bessel eigenfunction with sine and cosine angular dependence (|n′| = 2). Very close zeros of these Bessel functions (red oval in the right bottom panel) determine almost equal values of the radial wave vectors, which in turn leads to the almost equal frequencies, i.e., the second mode is degenerated three times. In the same way, the third mode (blue letters) contains five Bessel eigenfunctions with correspondent indices (see text). The right top panel: splitting of the degenerated modes described above in the slightly canted magnetic field. The right bottom panel: zeros of Bessel functions, which determine the radial wave vectors, are divided into groups (by black, red and blue ovals), corresponding to the three first split modes.

The second term in Eq. (2), proportional to sin^2^(*θ*_0_), gives deviations from the formula (3) due to a small inclination of the external field and magnetization from the perpendicular direction. This term is the only one “responsible” for the interaction between eigenfunctions of different numbers *n* and *n*΄, playing a role of the non-diagonal matrix element. Due to the special symmetry of the dipole-dipole operator, this interaction is non-zero only in the case when *n*΄ = *n* ± 2, see ref. [1]. The roots of the Bessel functions with such numbers *n* and *n*΄ coincide in the limit of large arguments. However, it is evident that already the values of the second root of *J*_2_(*ρ*) and second root of *J*_0_(*ρ*) are quite close to each other (see Fig. 6, red oval). As a consequence, the diagonal terms *ω*_*s*_(*θ*_0_)^2^ with *k* = *k*_02_ (corresponding to the SW profile *m*_02_(*ρ*, *φ*)) and with *k* = *k*_22_(spin wave profile *m*_22_(*ρ*, *φ*)) are found to be almost equal for all angles *θ* used in the experiment. In such a case only the non-diagonal matrix element in Eq. (2) gives the difference between the energies of the modes with *n* = 0 and *n΄* = ±2 (splitting).

As a result, we propose the following scenario of mode splitting. The results of the corresponding calculations are presented in Fig. 4 by solid lines. The first root of the Bessel function with *n* = 0 has no neighbors, and so, the lowest mode *i* = 1 does not split (see the black curve on the top graph and black oval on the bottom graph of Fig. 6). The second mode, *i* = 2, which contains eigenfunctions with the numbers *n* = 0 and *n΄* = ±2, should split into three levels (red curve on the top graph and red oval on the bottom graph of Fig. 6). The third mode *i* = 3 is a “mix” of five eigenfunctions (*n*΄ = 0, ±4, *n* = ±2), and so, it should split into five levels, etc.

## Discussion

The proposed theory can quantitatively describe the experimental results if both angles *θ* and *θ*_0_ are small. The absence of the full coincidence between theory and experiment in Fig. 3 can be associated with the effect of the dynamical magnetization pinning studied recently in^16^. Here for the simplicity the pinned boundary conditions were used for all the cases, when it has been proven in refs. 17 and 18 that the dipolar boundary conditions depend on the angle between the variable magnetization **m** and the boundary of the dot. This means that the boundary conditions depend on the angle *θ*_0_ that was not taken into account in the proposed theory. Secondly, the perturbation theory used here is valid when the energy gaps between the mode levels of different numbers are noticeable. However, as it is shown in Fig. 6, the levels of splitting modes become close to each other or even cross near *θ* ≈ 5°, which evidently restrict the validity of the perturbation theory for larger angles. Despite the mentioned issues, the difference between the experimental and theoretical results is below 5% even for *θ* = 5°.

Summarizing, in this article we investigated the spectra of standing spin waves in arrays of planar soft ferromagnetic dots with a circular shape when the external magnetic field is deviated from the direction perpendicular to the dots plane. Is such a case the cylindrical symmetry of the isolated dot is violated. Our experimental results unambiguously show that in such a case the lowest mode does not split, the second mode splits into 3 levels and the third one splits into 5 levels. The phenomenological perturbation theory developed here explains such behavior and gives a simple rule that defines the number of levels that corresponds to the mode number in the symmetrical case as 2*i* – 1. The profile of each mode is a combination of Bessel functions of even order.

## Methods

### Sample fabrication

Sample was fabricated by means of the electron-beam lithography and lift-off technique using a Raith 150 electron-beam writer. Py film with a thickness *of* 40 nm was deposited using molecular-beam epitaxy in a vacuum of 10^–8^ mbar. To improve the magnetic film quality, a 2 nm thick Cr underlayer was deposited on the substrate prior to the Py growth. To protect the Permalloy film, it was covered by a 2 nm thick Al layer, that fully oxidized after exposure to the atmosphere.

### Ferromagnetic resonance measurements

Room temperature FMR measurements were done at 9.85 GHz using standard electron spin resonance spectrometers Bruker ELEXSYS E500 and Bruker ESP 300. In both cases a computer-controlled goniometer allowed varying the angle *θ* between the external magnetic field ***H*** and the sample normal to the array plane ***n*** with an accuracy of 0.1^o^.

## Additional Information

**How to cite this article**: Bunyaev, S. A. *et al.* Splitting of standing spin-wave modes in circular submicron ferromagnetic dot under axial symmetry violation. *Sci. Rep.* **5**, 18480; doi: 10.1038/srep18480 (2015).
